# Assessment of the Microbiological Safety and Hygiene of Raw and Thermally Treated Milk Cheeses Marketed in Central Italy between 2013 and 2020

**DOI:** 10.3390/life13122324

**Published:** 2023-12-11

**Authors:** Sara Primavilla, Rossana Roila, Elena Rocchegiani, Giuliana Blasi, Annalisa Petruzzelli, Claudia Gabucci, Donatella Ottaviani, Stefania Di Lullo, Raffaella Branciari, David Ranucci, Andrea Valiani

**Affiliations:** 1 Istituto Zooprofilattico Sperimentale dell’Umbria e delle Marche “Togo Rosati”, Via Salvemini 1, 06126 Perugia, Italy; s.primavilla@izsum.it (S.P.); e.rocchegiani@izsum.it (E.R.); g.blasi@izsum.it (G.B.); a.petruzzelli@izsum.it (A.P.); c.gabucci@izsum.it (C.G.); d.ottaviani@izsum.it (D.O.); s.dilullo@izsum.it (S.D.L.); a.valiani@izsum.it (A.V.); 2 Department of Veterinary Medicine, University of Perugia, Via San Costanzo 4, 06126 Perugia, Italy; raffaella.branciari@unipg.it (R.B.); david.ranucci@unipg.it (D.R.)

**Keywords:** foodborne infection, food safety, cheese, *Listeria monocytogenes*, *Salmonella* spp., staphylococcal enterotoxin, *Escherichia coli*, coagulase-positive staphylococci

## Abstract

A profile of the microbial safety and hygiene of cheese in central Italy was defined based on an analysis of 1373 cheeses sampled under the Italian National Control Plan for Food Safety spanning the years 2013 to 2020 and tested according to Commission Regulation (EC) No. 2073/2005 (as amended). A total of 97.4% of cheese samples were assessed as being satisfactory for food safety criteria and 80.5% for process hygiene criteria. Staphylococcal enterotoxin was found in 2/414 samples, while *Salmonella* spp. and *Listeria monocytogenes* were detected in 15 samples out of 373 and 437, respectively. *Escherichia coli* and coagulase-positive staphylococci counts were found unsatisfactory in 12/61 and 17/88 cheese samples, respectively. The impact of milking species, milk thermal treatment, and cheese hardness category was considered. A statistically significant association (*p* < 0.05) was found between milk thermal treatment and the prevalence of coagulase-positive staphylococci and *Listeria monocytogenes* and between hardness and unsatisfactory levels of *Escherichia coli*. The data depict a contained public health risk associated with these products and confirm, at the same time, the importance of strict compliance with good hygiene practices during milk and cheese production. These results can assist in bolstering risk analysis and providing insights for food safety decision making.

## 1. Introduction

Cheese represents the most diverse group of dairy products obtained, with a wide range of tastes, flavors, and forms throughout the world [1]. Although the primary objective of cheese making was to conserve the principal constituents of milk, cheese has evolved to become a largely appreciated food with elevated culinary qualities, as well as nutritional ones [2]. The high consumer acceptability of cheeses can be attributed to their pleasant sensorial characteristics, good nutritional properties, versatility of use, and the introduction of novel ingredients, packaging, and sale formats [3,4]. Cheeses are generally considered a safe food, although, unlike other products, they are biologically and biochemically dynamic and, consequently, characterized by an inherent instability [1]. The safety of cheese depends upon a variety of factors influencing the growth, survival, and inactivation of microorganisms, such as the microbiological quality of the raw milk, the rate and degree of acidification during production, and the water activity (a_w_) of the final product.

Although raw milk is often identified as the primary contributor to cheese contamination, it is not the sole source [5], and the ability of some pathogens to form biofilm and persist on food contact surfaces has been related to cross-contamination during and after production [6,7].

Several factors can influence cheese microbial safety and hygiene, such as the origin and microbial quality of milk, the adjustment of fat content, milk homogenization, pasteurization, starter cultures, coagulation, curd manipulation, hand stirring, salting, whey removal, milling, molding, and storage conditions [8]. For instance, a worker’s hands and cheese contact surfaces in processing plants have been identified as potential vehicles for some harmful microorganisms [9], such as *Staphylococcus aureus* [10,11] and *Listeria monocytogenes* [12]. Furthermore, once contamination has occurred, the intrinsic characteristics of cheese can potentially allow for microbial growth.

Consequently, numerous foodborne illnesses linked to cheese consumption have been reported over the years in many countries [13].

The European Food Safety Authority (EFSA) states that in 2020, 1.6% of foodborne outbreaks in the European Union were associated with cheeses, albeit data from previous years revealed even higher values, such as 7.8% in 2015 and 4.8% in 2016 [13].

One of the main concerns among food manufacturers, retailers, and researchers, as well as regulatory and official control agencies, is to ensure consumers safe cheeses.

In European countries, cheese production and sales are subjected to Regulation (EC) No. 2073/2005 [14], amended by Regulation (EC) No. 1441/2007 [15], and microbiological criteria, namely, food safety criteria and process hygiene criteria, vary according to the microorganism of concern.

The objective of this study is to compile findings from the Italian National Control Plan for Food Safety spanning the years 2013 to 2020 in order to create a comprehensive overview of the microbiological safety and hygiene status of cheese in central Italy in accordance with the microbiological criteria outlined in Regulation (EC) No. 2073/2005 [14].

## 2. Materials and Methods

### 2.1. Sample Collection and Description of Samples

Cheese samples were collected by veterinary inspectors in the Umbria and Marche regions (central Italy) between 2013 and 2020, in the context of the Italian National Control Plan for Food Safety. Finished products were sampled in dairy factories and retail stores. The cheeses were classified according to the type of milk used (ovine or bovine cheese), heat treatment (raw milk or pasteurized milk), and hardness category (fresh, soft/semisoft, and hard/semihard) [16]. Samples were aseptically collected using sterile instruments and sterile bags (Blender bag Plain, 400 mL, Sterile VWR^®^, Milano, Italy) and stored at refrigerated temperature (+4 °C) until analyzed, according to UNI EN ISO 7218:2013 [17]. Each sample included five subunits of at least 100 g, which were randomly collected from each lot of sampled cheese. The collected samples were stored at refrigerated temperature (+4 °C) and analyzed within 24 h.

### 2.2. Sample Assessment

The analysis pattern of each sample was performed according to the Italian National Control Plan for Food Safety; therefore, not all samples were subjected to the same microbiological determinations.

Test results were assessed as being satisfactory or unsatisfactory using the assessment criteria outlined in Table 1, which are based on Regulation (EC) No. 2073/2005 [14], amended by Regulation (EC) No. 1441/2007 [15]. In particular, Table 1A shows the interpretation of the results for food safety criteria (*Salmonella* spp. *L. monocytogenes* and Staphylococcal enterotoxins), as described in Chapter 1 of the Regulation. Table 1B, instead, reports the interpretation of the results for process hygiene criteria (enumeration of *Escherichia coli* and coagulase-positive staphylococci), as shown in Chapter 2, Section 2.2 “Milk and dairy products” of the Regulation [14,15].

### 2.3. Microbiological Analysis

Microbiological tests were performed in UNI CEI EN ISO/IEC 17025:2018 [18] accredited food microbiology laboratories of “Istituto Zooprofilattico Sperimentale dell’Umbria e delle Marche Togo Rosati”.

Qualitative analyses (*Listeria monocytogenes*—LMO, *Salmonella* spp.—SLM, and Staphylococcal enterotoxins—SEs) were performed through an enzyme-linked fluorescent immunoassay (VIDAS^®^ LMO, VIDAS^®^ SLM, and VIDAS^®^ SET2 tests, respectively—bioMérieux, Marcy-I’Etoile, France), following the instructions provided by the manufacturer. In brief, for LMO and SLM detection, a 10^−1^ homogenate of each sample was prepared by diluting 25 g of cheese in 225 mL of Half Fraser Broth (Microbiol s.r.l., Cagliari, Italy) and Buffered Peptone Water (Biolife s.r.l., Milan, Italy), respectively. Positive broth cultures were confirmed according to ISO 11290-1:2017 [19] for LMO and to ISO 6579-1:2017 [20] for SLM. LMO was confirmed using two different culture media, ALOA (Ottaviani Agosti Listeria agar) and Oxford agar, both purchased from Microbiol s.r.l., Cagliari, Italy. Blue-green colonies with an opaque halo on ALOA and brown colonies with black aesculin hydrolysis zones on Oxford agar were selected for further tests (API Listeria—bioMérieux, Marcy-I’Etoile, France). Regarding SLM, two different selective and differential media were used: xylose–lysine–deoxycholate agar (XLD—Microbiol s.r.l., Cagliari, Italy) and Salmonella chromogenic medium (Biolife Italiana s.r.l., Milan, Italy). Typical colonies (red with a black center on XLD agar and magenta on Salmonella chromogenic medium) were chosen to be confirmed through biochemical tests (API 32E strips—bioMérieux, Marcy-I’Etoile, France). SLM isolates were finally serotyped through a slide agglutination test, as described in the White–Kauffmann–Le Minor scheme [21,22].

SE detection was performed according to ISO 19020:2017 [23]: 25 g of each cheese sample was homogenized with 40 mL of preheated (38 ± 2 °C) demineralized water. After 30 min at 18–25 °C, the pH of the homogenized samples was adjusted between 3.5 and 4.0 using 5 N HCl. The suspension was then centrifuged for 15 min at 3130× *g* at 4 °C. The supernatant was recovered, and the pH was adjusted between 7.4 and 7.6 using 1 N NaOH. The suspension was finally centrifuged for 15 min at 4 °C at 3130× *g* and placed in a dialysis sac that was immersed in 30% (*w/v*) polyethylene glycol (PEG; Sigma-Aldrich, St. Louis, MO, USA) at 5 °C until the volume was reduced to 15–20 mL or less. The sac was then rinsed inside with phosphate-buffered saline (PBS; pH, 7.3 ± 0.2; NaCl, 145 mM; Sigma-Aldrich, St. Louis, MO, USA—Na_2_HPO_4_, 10 mM; Chem-Lab NV, Zedelgem, Belgium), and 500 μL was loaded into Vidas^®^ SET2 strips. The results of qualitative analyses were expressed as “presence” or “non-detection” of the pathogen/toxin in 25 g of sample.

Enumeration of LMO was performed as described in ISO 11290-2:2017 certification [24]: 10 g of each sample was properly homogenized with 90 mL of Buffered Peptone Water (Biolife Italiana s.r.l., Milan, Italy), serially 10-fold diluted and spread onto ALOA. Plates were incubated at 37 °C for 24–48 h before counting typical LMO colonies (blue-green colonies surrounded by an opaque halo). A maximum of five or fewer suspect colonies from each plate were then confirmed using biochemical or molecular tests, as described in ISO 11290-2:2017 [24], and the final count was determined in proportion to the initial number.

Enumeration of *Escherichia coli* (EC) and coagulase-positive staphylococci (CPS) was carried out utilizing a TEMPO^®^ automated enumeration system (bioMérieux, Marcy-I’Etoile, France), starting from samples properly diluted in Buffered Peptone Water (Biolife Italiana s.r.l., Milan, Italy).

The results of quantitative analyses were expressed as colony-forming units/gram (CFU/g).

### 2.4. Criteria for the Interpretation of Microbiology Results

Microbiological results were interpreted using Commission Regulation (EC) No. 2073/2005 (as amended) [14,15]. As summarized in Table 1A,B, this Regulation defines limits for SE, SLM, LMO, and CPS. However, it does not provide criteria for EC in unpasteurized milk cheese, even though it sets limits for this organism in cheese made from pasteurized milk.

### 2.5. Statistical Analysis

Descriptive and statistical analyses of the data were performed using R free software version 4.3.2 (R Foundation for Statistical Computing, Vienna, Austria). Relative proportions were compared using the Chi-squared and Fisher–Yates test to determine if there were associations between the assessment status of the samples (i.e., satisfactory or unsatisfactory) and sample variables (i.e., milking species, thermal treatment, hardness cheese category, and year of sampling). For the purpose of this analysis, *p*-values < 0.05 were deemed to be statistically significant.

## 3. Results and Discussion

**Figure 1:**
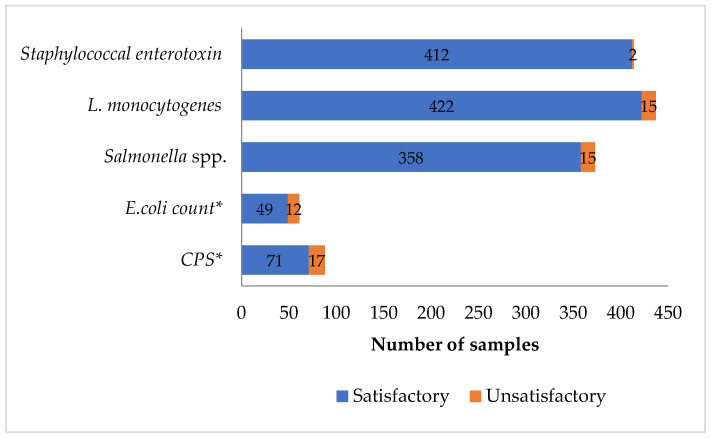
Graphical representation of microbiological results from testing of raw and thermally treated milk cheeses. * Satisfactory categorization includes acceptable samples; CPS—coagulase-positive staphylococci.

Figure 1 shows a comprehensive profile of microbial characteristics of cheese sampled in central Italy from 2013 to 2020 in the context of the Italian National Control Plan for Food Safety. When interpreted according to the criteria shown in Table 1, 1312 determinations (95.6%) were considered to be microbiologically satisfactory, whereas 61 (4.4%) were unsatisfactory. In detail, concerning the food safety criteria, the cheese samples interpreted as unsatisfactory and potentially harmful were found positive, in order, for *Salmonella* spp. (4%; 15/373), followed by *L. monocytogenes* (3.4%; 15/437) and Staphylococcal enterotoxins (0.5%; 2/414). Pertaining to the process hygiene criteria, 29/149 samples (19.5%) were reported as unsatisfactory with a slightly higher prevalence of EC levels (19.7%; 12/61) than CPS (19.3%; 17/88). Although the presence of pathogenic bacteria or toxins has been reported, the overall results reveal a satisfactory microbiological hygiene and safety profile of the cheese samples collected in central Italy. In terms of unsatisfactory samples, the herein reported values are lower than those registered in England for the years 2013–2020 (12%), albeit the latter considered only unpasteurized milk cheese [2]. Furthermore, another English study revealed that results from routine monitoring were unsatisfactory for 21% of samples, considering cheeses from different species [25]. Ganz and colleagues refer to an overall value of samples assessed as being of unsatisfactory microbiological safety of 2.2% for different cheeses sampled in Canada during 2012–2017 [26], which is lower but still comparable to the value herein reported.

Statistical analysis performed with the Chi-square test indicated no significant relationship between the year of sampling and the frequency of unsatisfactory samples in individual years (*p* ≥ 0.05); therefore, this variable has not been considered in this study. Any differences attributable to the considered milking species, the application of heat treatments in milk, and the cheese hardness category were taken into account and further discussed.

### 3.1. Food Safety Criteria

#### 3.1.1. *Listeria monocytogenes*

*L. monocytogenes*, a well-known foodborne pathogen, has the potential to cause serious and life-threatening conditions, especially for the immunocompromised, such as septicemia, meningitis, stillbirths, and abortion [27]. *L. monocytogenes* is widespread in the environment, and it is able to enter food processing plants, particularly dairy ones, where it can easily establish itself and strongly persist thanks to its ability to form biofilms and manifest decreased susceptibility to disinfectants and the environment [28].

According to the data depicted in the present study, *L. monocytogenes* was detected in bovine and ovine milk cheeses with similar prevalences of 3.88% and 3.25%, respectively (Table 2), indicating that milk-producing species seems to be irrelevant for the presence of this pathogen in cheese (*p* > 0.05).

Different results were highlighted in relation to milk thermal treatment, revealing that raw milk cheeses show a lower prevalence of contamination compared to pasteurized milk cheeses (Table 3). This result may seem unlikely since pasteurized milk is generally recognized as safer than raw milk [25]; however, this outcome is in line with the literature data reporting that even though pasteurization inactivates *L.monocytogenes* in raw milk, pasteurized milk cheeses are still a major source of *L. monocytogenes* infection [29,30].

This is primarily due to insufficient pasteurization or post-pasteurization cross-contamination that can occur in almost any phase of the production and distribution chain in relation to its ability to persist in dairy production environments [29,31]. Some authors [32] have also reported that post-processing handling and operations constitute a major concern for *L. monocytogenes* contamination; for instance, cheese cutting or slicing could represent a critical point for this microorganism being transferred to the paste, where it can easily grow and, in some cases, exceed the limit imposed by food safety criteria of Regulation (EC) No. 2073/2005 [14].

Furthermore, it has been reported that natural raw milk microbiota may be able to prevent the growth of contaminating foodborne pathogens during cheese making, defining raw milk cheeses as a rather microbiologically safe food. For instance, extremely low or zero percentages of raw milk cheeses contaminated with *L. monocytogenes* have been reported in the literature [8,33,34].

Albeit the prevalence values related to the hardness category seem to suggest a higher involvement of harder cheese (Table 4), these values are not significant (*p* > 0.05). The reported prevalence of *L. monocytogenes* in fresh cheeses from different countries is quite variable, ranging from 0.0% to 38%, even if the majority are below 10% [8,35,36]. For ripened cheese, the values registered in different countries range from 0.0% to 5.5% [37], which is in line with the results shown in this study. It has been reported in the literature that *L. monocytogenes* presence in milk and dairy products can be significantly influenced by geographical area and seasons [38]; however, these two aspects are not considered in the present study.

#### 3.1.2. *Salmonella* spp.

All Salmonella strains are gastroenteritis-inducing pathogens, and despite the implementation of integrated and harmonized control programs, this pathogen still represents a significant cause of foodborne outbreaks in Europe [39].

Various Salmonella serotypes have been involved in cheese-borne outbreaks [40]. For instance, a nationwide *Salmonella* outbreak in Dublin associated with raw cow milk cheese consumption was investigated in France in 2015–2016 [41], and strong evidence of an outbreak of *Salmonella* Enteritidis caused by the consumption of contaminated raw sheep milk cheese was more recently deepened in central Italy [42].

As reported above, the mean prevalence of this pathogen in sampled cheeses during the time frame considered was 4.00%, which is primarily attributable to ovine milk cheeses, indicating, as shown in Table 2, that the related prevalence of this pathogen was higher than bovine milk cheese, albeit with no statistical significance (*p* > 0.05). Concerning milk thermal treatment and hardness categories, the data reported in Table 3 and Table 4 suggest (*p* > 0.05) the major involvement of raw milk cheeses, as the entire amount of positive samples belonged to this latter category, and of fresh or soft and semisoft cheese, as in these two cases, the prevalence is double the hard and semihard cheeses. These results are partially in agreement with the data available in the literature. Elafify et al. reported prevalence values of 16.7% for Kariesh cheese (a ewe milk soft fresh cheese) from retailed dairies in Egypt [43]. A meta-analysis of data from different countries revealed that the pooled frequency of detection of *Salmonella* in sheep milk cheese was 1.4%, which is higher than in our study [44]. Another work from Costanzo and colleagues revealed a 0% prevalence of contamination with Salmonella in both ovine and bovine raw milk cheese from southern Italy [36]. Spanish authors highlight the importance of the presence of antibiotic-resistant Salmonella species in food samples, although the prevalence they registered in pasteurized milk cheese was 0% (0/289) [45].

#### 3.1.3. Staphylococcal Enterotoxins

The ingestion of Staphylococcal enterotoxins can cause food poisoning outbreaks, with common symptoms being vomiting, diarrhea, nausea, and abdominal pain, which appear after a short incubation period (2–6 h). Just a few micrograms of Staphylococcal enterotoxins are sufficient to cause food poisoning in vulnerable adults, while nanograms are enough in children [46].

Staphylococcal enterotoxins produced by *Staphylococcus aureus* are, on a global scale, one of the most common causative agents of food poisoning [13]; indeed, it has also been reported that in 2015, over half of the foodborne outbreaks associated with bacterial toxins (n = 434) were caused by Staphylococcal enterotoxins [47]. In the literature, several outbreaks of staphylococcal food poisoning due to the consumption of cheese have been reported [48,49,50].

As previously mentioned, in the present study, the overall mean prevalence of SEs in cheese was 0.5% (2/414), indicating a low concern for public health related to the presence of this toxic contaminant, as higher values have been registered in the literature. For instance, the European Food Safety Authority (EFSA) in 2017 reported that approximately 1.2% of cheeses and dairy products, analyzed in the context of national monitoring and surveillance plans of reporting member states, tested positive for staphylococcal enterotoxin [51]. Other studies from Turkey reported values of 2.3% and 3.8% for Staphylococcal enterotoxin-positive samples of ewe cheese and dairy dessert, respectively [52].

Among the 414 determinations for Staphylococcal enterotoxins, only 2 were found positive, referring to the samples of cheese from raw ovine milk, of which one was fresh and one was soft and semisoft. Table 2, Table 3 and Table 4 show no significant differences (*p* > 0.05) among milk-producing species, milk thermal treatment, or hardness category, respectively, and due to the low prevalence values, scarce discussion can be conducted. Several methods can limit the growth of *Staphylococcus aureus* and its enterotoxin production during cheesemaking, such as the use of starter cultures, high salt concentration, low storage temperature, and extreme pH [53].

### 3.2. Process Hygiene Criteria

#### 3.2.1. *Escherichia coli* count

Coliforms are not inherent microflora of raw milk; thus, their presence in cheese can be indicative of milk fecal contamination stemming from the environment, udder, and milking equipment during and after the milking process [54]. Additionally, it is noteworthy that ruminants serve as a reservoir for *E. coli*, and defecation during the milking procedure is recognized as a pivotal event with the potential for milk contamination. Therefore, the rigorous maintenance of robust milking and hygiene protocols is imperative in mitigating these risks of microbial contamination [55]. For more than a century, the dairy sector has monitored sanitation failures and post-processing contamination using these hygiene indicators, and nowadays, many industrialized and developing nations throughout the world have set microbial indicator thresholds for assessing the hygienic quality of cheeses [56]. Consistent with EU Regulation 2073/2005, in this study, *E. coli* was considered as an indicator of the level of process hygiene [14]. Of 61 determinations in cheese samples, 12 were above the regulatory limit, with an overall prevalence of 19.67% (95%).

Concerning the milk-producing species, the prevalence of unsatisfactory samples was equivalent, with 21% for bovine and 16% for ovine (Table 2). Previous results from the literature have reported lower values for *E. coli* contamination in southern Italy, at 7.6% and 3.7% for ovine and bovine milk cheeses, respectively [36].

Concerning the results related to milk thermal treatment, it is important to highlight that national sampling plans are defined on the basis of the regulatory framework that stipulates that the enumeration of *E. coli* shall only be conducted on samples of heat-treated milk cheese. Therefore, no data are available on raw milk cheeses; therefore, a comparison with pasteurized samples is unfeasible.

According to the hardness of cheese, the prevalence of non-compliant samples was lower for fresh cheese compared to those of ripened cheeses (*p* < 0.05), although the value for hard and semihard cheese aligns with both previous categories.

#### 3.2.2. Coagulase-Positive Staphylococci

Staphylococcal food poisoning is one of the most frequent foodborne illnesses in humans worldwide and is associated with contaminated foods of animal origin, such as milk and dairy products and other protein-rich foods of animal origin [57,58].

*Staphylococcus aureus* subsp. *aureus*, the representative species of coagulase-positive staphylococci (CPS), frequently colonizes the ductus papillaris of the mammary glands in milk-producing animals, which can lead to the recurrent occurrence of clinical or subclinical mastitis. This causes the frequent use of antibiotics in the dairy sector and explains the presence of this microorganism in milk [2]. Furthermore, the environment, including human handling, can serve as a significant source of contamination; consequently, it is highly advisable to enhance hygiene practices during production [34]. Many studies in the literature have shown that 15% to 80% of the CPS isolated from various food matrices are able to produce thermo-stable and pepsin-resistant enterotoxins, especially when the bacterial load exceeds 10^5^ CFU/g [36,59], justifying the relevance of this process hygiene criterion strongly related to food safety.

The overall data, interpreted according to the criteria shown in Table 1, show that 17 samples out of 88 (19.3%) were unsatisfactory, which is similar to what was previously reported by other authors [60].

Our results for the two milk-producing species considered suggest a higher prevalence in ovine samples (Table 2), although without statistical significance (*p* > 0.05).

Furthermore, the data reported in Table 3 demonstrate that raw milk cheese has a higher prevalence of CPS compared to pasteurized milk cheese (*p* < 0.05). The widespread occurrence of CPS in raw milk cheese is a widely recognized fact [61], and our results align with those of other authors who reported a higher prevalence in this matrix [62]. From these results, it can be inferred that during the production of cheese, the pasteurization of milk is an effective control measure to reduce the exposure of consumers to this class of potentially harmful microorganisms and their toxins [63]. Although hard and semihard cheese showed a 0% prevalence (Table 4), these data were not significantly lower than those of fresh or soft and semisoft cheese (15% and 25%, respectively), failing to statistically prove that harder cheese paste is associated with lower CPS prevalence.

## 4. Conclusions

Poor sanitation and a lack of hygienic practices during milk handling and processing, as well as animal health issues, can be considered potential sources for the microbial contamination of raw milk and, potentially, cheese. Although pasteurization effectively eliminates most unwanted microorganisms present in raw milk, the potential for foodborne illnesses also remains a concern for heat-treated milk cheeses, which is due to faulty processing or post-processing contamination caused by the establishment and persistence of foodborne pathogens in dairy processing facilities.

Although few incompliant samples have been registered, overall, the microbiological analyses performed on different cheese types in central Italy during 2013–2020 and depicted in the present study show satisfactory levels of microbiological safety and hygiene of these products. No remarkable differences in the prevalence of unsatisfactory samples have been highlighted considering the milk-producing species. The milk heat treatment seems to affect the microbiological characteristics of cheese in different ways, as this is related to the higher prevalence of *L. monocytogenes* and the lower counts of CPS. Concerning the hardness of cheese paste, the results highlight its effect in decreasing *E. coli* count.

The reported prevalence of pathogenic microorganisms in raw and pasteurized milk cheese is contained. Nevertheless, the various outbreaks registered around the world, which can be attributed to the presence of these bacteria in cheeses, indicate the importance of control measures to be adopted in every step of cheese production and commercialization according to the European laws in force and following the National Control Plans.

## Figures and Tables

**Table 1:** Microbiological assessment criteria used to assess the microbiological safety (A) and quality (B) of cheese analyzed under the National Microbiological Monitoring Program and Targeted Survey Program.

**A**								
**Food Safety Criteria**	**Cheese Category**						**Results Interpretation**	
							**Satisfactory**	**Unsatisfactory**
*L. monocytogenes*	Cheeses able to support the growth of L. monocytogenes *						Not detected in 25 g of each of the 5 sample units	Presence in 25 g in any of the 5 sample units
	Cheeses unable to support the growth of L. monocytogenes *						<100 cfu/g in each of the 5 sample units	>100 cfu/g in any of the 5 sample units
*Salmonella* spp.	Cheeses made from raw milk or milk that has undergone a lower heat treatment than pasteurisation						Not detected in 25 g of each of the 5 sample units	Presence in 25 g in any of the 5 sample units
Staphylococcal enterotoxins	Cheeses from raw milk, cheeses made from milk that has undergone a lower heat treatment than pasteurisation and ripened cheeses made from milk or whey that has undergone pasteurisation or a stronger heat treatment and unripened soft cheeses (fresh cheeses) made from milk or whey that has undergone pasteurisation or a stronger heat treatment						Not detected in 25 g of each of the 5 sample units	Presence in 25 g in any of the 5 sample units
**B**								
**Process Hygiene Criteria**	**Cheese Category**	**Sampling Plan**		**Limits** **(cfu/g)**		**Results Interpretation**		
		**n**	**c**	**m**	**M**	**Satisfactory**	**Acceptable**	**Unsatisfactory**
*E. coli*	Cheeses made from milk or whey that has undergone heat treatment	5	2	100	1000	All the values observed are ≤m	A maximum of c/n values are between m and M, and the rest of the values observed are ≤m	One or more of the values observed are >M or more than c/n values are between m and M
CPS	Cheeses made from raw milk	5	2	10^4^	10^5^			
	Cheeses made from milk that has undergone a lower heat treatment than pasteurisation and ripened cheeses made from milk or whey that has undergone pasteurisation or a stronger heat treatment	5	2	100	1000			
	Unripened soft cheeses (fresh cheeses) made from milk or whey that has undergone pasteurisation or a stronger heat treatment	5	2	10	100			

n—number of units comprising the sample; c—number of sample units giving values between m and M; m—limit value within which the result is acceptable; M—limit value above which the result is not acceptable; CPS—coagulase-positive staphylococci. * The criteria for determining whether or not a product supports the growth of *L. monocytogenes* are described in Regulation (EC) No. 2073/2005 [14], amended by Regulation (EC) No. 1441/2007 (Chapter 1—Note 8) [15].

**Table 2:** Unsatisfactory cheese samples tested under the National Control Plan for Food Safety according to the milking species.

	Bovine Milk Cheese				Ovine Milk Cheese			
	Tot.	Unsatisfactory	Prevalence	95% CI	Tot.	Unsatisfactory	Prevalence	95% CI
Staphylococcal enterotoxin	116	0	0		298	2	0.67	0.16–1.18
*L. monocytogenes*	129	5	3.88	0.95–7.81	308	10	3.25	1.53–5.97
*Salmonella* spp.	85	1	1.23	0.00–2.95	288	14	4.86	2.97–6.75
*E. coli* count	42	9	21.43	11.49–31.37	19	3	15.79	7.88–23.70
CPS	31	3	9.68	3.90–15.46	57	14	24.56	15.41–33.71

95% CI—95% confidence interval.

**Table 3:** Unsatisfactory cheese samples tested under the National Control Plan for Food Safety according to milk thermal treatment.

	Raw Milk Cheese				Pasteurized Milk Cheese			
	Tot.	Unsatisfactory	Prevalence	95% CI	Tot.	Unsatisfactory	Prevalence	95% CI
Staphylococcal enterotoxin	316	2	0.63	0.14–2.13	99	0	0	0
*L. monocytogenes*	290	6	2.07 a	0.88–4.37	147	8	5.44 b	2.34–10.56
*Salmonella* spp.	303	15	4.95	2.89–7.94	70	0	0	0
*E. coli* count	-	-	-	-	61	12	19.67	11.29–29.53
CPS	55	15	27.27 b	15.89–39.64	33	2	6.06 a	0.75–19.99

Different letters in the same row (a,b) indicate differences between prevalence values (*p* < 0.05); 95% CI—95% confidence interval.

**Table 4:** Unsatisfactory cheese samples tested under the National Control Plan for Food Safety according to cheese hardness category.

	Fresh Cheese			Soft and Semisoft Cheese			Hard and Semihard Cheese		
	Tot.	Unsatisfactory	Prevalence (95% CI)	Tot.	Unsatisfactory	Prevalence (95% CI)	Tot.	Unsatisfactory	Prevalence (95% CI)
Staphylococcal enterotoxin	110	1	0.91 (0.02–4.80)	169	1	0.59 (0.01–3.32)	135	0	0
*L. monocytogenes*	108	1	0.93 (0.02–4.94)	182	8	4.40 (1.90–8.47)	147	6	4.08 (1.49–8.73)
*Salmonella* spp.	102	6	5.88 (2.13–12.42)	143	6	4.20 (1.70–9.10)	128	3	2.34 (0.48–6.74)
*E. coli* count	22	1	4.54 a (0.11–22.63)	18	6	33.33 b (14.95–58.19)	21	5	23.81 ab (9.28–44.46)
CPS	20	3	15.00 (3.19–36.19)	56	14	25.00 (14.52–37.39)	12	0	0

Different letters in the same row (a,b) indicate differences between prevalence values (*p* < 0.05); 95% CI—95% confidence interval.

## Data Availability

Data are available from the authors.
